# A cross-sectional study evaluating cardiovascular risk and statin prescribing in the Canadian Primary Care Sentinel Surveillance Network database

**DOI:** 10.1186/s12875-022-01735-6

**Published:** 2022-05-25

**Authors:** Ian S. Johnston, Brendan Miles, Boglarka Soos, Stephanie Garies, Grace Perez, John A. Queenan, Neil Drummond, Alexander Singer

**Affiliations:** 1 University of Calgary, Calgary, Canada; 2 Crowfoot Village Family Practice, Suite 210, 600 Crowfoot Crescent NW, Calgary, Alberta T3G 0B4 Canada; 3 Queens University, Kingston, Canada; 4 University of Alberta, Edmonton, Canada; 5 University of Manitoba, Winnipeg, Canada

**Keywords:** Primary care, Cardiovascular disease, Electronic medical records, Socioeconomic deprivation

## Abstract

**Background:**

Cardiovascular disease (CVD) is a major cause of morbidity and mortality in Canada. Assessment and management of CVD risk is essential in reducing disease burden. This includes both clinical risk factors and socioeconomic factors, though few studies report on socioeconomic status in relation to CVD risk and treatment. The primary objective of this study was to estimate the cardiovascular risk of patients attending primary care practices across Canada; secondly, to evaluate concordance with care indicators suggested by current clinical practice guidelines for statin prescribing according to patients’ cardiovascular risk and socioeconomic status.

**Methods:**

This cross-sectional observational study used the Canadian Primary Care Sentinel Surveillance Network (CPCSSN) database, which is comprised of clinical data from primary care electronic medical records. Patients aged 35-75y with at least one visit to their primary care provider between 2012 and 2016 were included. Patients were assigned to a CVD risk category (high, medium, low) and a deprivation quintile was calculated for those with full postal code available. Descriptive analyses were used to determine the proportion of patients in each risk category. Logistic regression was used to evaluate the consistency of statin prescribing according to national clinical guidelines by risk category and deprivation quintile.

**Results:**

A total of 324,526 patients were included. Of those, 116,947 (36%) of patients were assigned to a high CVD risk category, primarily older adults, males, and those with co-morbidities. There were statistically significant differences between least (quintile 1) and most (quintile 5) deprived socioeconomic quintiles, with those at high CVD risk disproportionately in Q5 (odds ratio 1.4). Overall, 48% of high-risk patients had at least one statin prescription in their record. Patients in the lower socioeconomic groups had a higher risk of statin treatment which deviated from clinical guidelines.

**Conclusions:**

Primary care patients who are at high CVD risk are more often male, older, have more co-morbidities and be assigned to more deprived SES quintiles, compared to those at low CVD risk. Additionally, patients who experience more challenging socioeconomic situations may be less likely to receive CVD treatment that is consistent with care guidelines.

**Supplementary information:**

The online version contains supplementary material available at 10.1186/s12875-022-01735-6.

## Background

Cardiovascular diseases (CVD) are significant causes of morbidity and mortality in Canada [1]. Fixed and modifiable risk factors such as age, gender, smoking, and diabetes are well known to affect individual risk of myocardial infarction and stroke [2]. There is evidence to suggest that lower socioeconomic status (SES) may contribute to a higher risk of CVD for individuals [3–5]. While the prevalence of CVD and its associated risk factors has been gradually increasing in Canada, the highest rates are observed among those of lower SES [6]. The importance of SES to CVD risk, including pharmacotherapy management, has been described in other countries such as the United Kingdom, [7, 8] but is not well studied in a Canadian primary care context.

The assessment and management of cardiovascular risk by primary care providers is a key component in reducing the population burden of CVD [9]. Current Canadian clinical guidelines recommend primary health care providers use a validated cardiovascular risk calculator to inform appropriate, patient-centered, management strategies to reduce CVD incidence, morbidity and mortality [2, 9, 10]. These guidelines also recommend that patients with known CVD should be offered statins for secondary prevention unless they experience adverse effects [2]. Targeting those individuals without evidence of established cardiovascular disease is considered primary prevention, whereas secondary prevention targets individuals with prior vascular events, or with evidence of known atherosclerotic vascular disease (Appendix A).

Despite these recommendations, an observational study in Manitoba demonstrated that many individuals who were prescribed statins had no history of CVD in their medical record (suggesting their use for primary prevention), and a majority of those with CVD were not prescribed statins as a secondary prevention measure [11]. This finding indicates a potential care gap and offers an opportunity to describe the potential effect of SES on prescribing appropriateness.

The primary objective of this study was to estimate the cardiovascular risk of patients attending primary care practices across Canada. The secondary objective was to evaluate concordance of current clinical practice guidelines for statin prescribing according to patients’ cardiovascular risk and socioeconomic status.

## Methods

### Data source

The pan-Canadian Primary Care Sentinel Surveillance Network (CPCSSN) is a collaboration of practice-based research networks which extract and process de-identified patient data from electronic medical records (EMR) in primary care [12]. The CPCSSN database includes key cardiovascular risk factor data, such as patient age, sex, smoking status, serum lipids, blood pressure, and diagnoses. CPCSSN uses validated case definitions to identify patients with common chronic conditions, including hypertension and diabetes [13]. The CPCSSN database and processes for cleaning and standardization have been described in detail elsewhere [12–15].

### Study design and participant selection

This was a cross-sectional study using national CPCSSN data up to December 31, 2016. Approximately 1.6 million patients of all ages are included in the database. For this study, patients aged 35–75 years with at least one visit to their primary care provider in the period January 1, 2012 to December 31, 2016 were included. Patients without a recorded birth year or gender, or without sufficient data to assign to a cardiovascular risk category, were excluded. CPCSSN data overall is considered to be reasonably representative of the Canadian base population, with slight skewing in favour of females and older adults [12, 14].

### Primary outcome measurement

**Fig.1:**
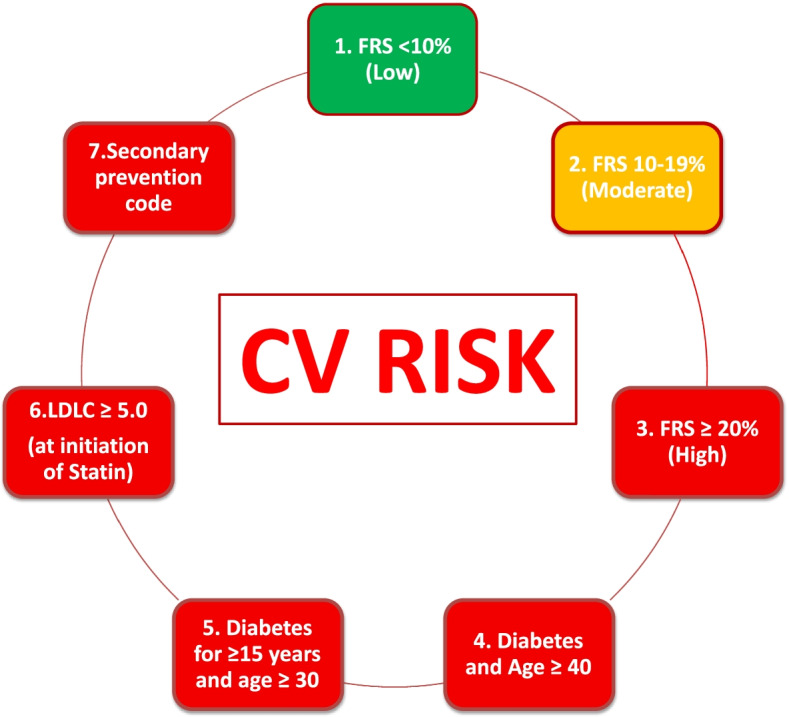
Risk groups as defined by CCS Guidelines2; those in red would warrant a statin recommendation/prescription

The primary study outcome was the proportion of patients at high, medium or low risk of developing CVD based on the 2016 Canadian Cardiovascular Society (CCS) guidelines (Fig. 1), reported by age group, sex (male or female), comorbid conditions (using validated definitions for diabetes mellitus and hypertension [13]), current smoking status and SES quintile. The Framingham Risk Score (FRS), which is recommended for use by the CCS, [2] was used to estimate 10-year CVD risk and facilitated the categorization of risk groups. Variables from the CPCSSN data used to calculate the FRS were patient age, sex, current smoking status, diagnosis of diabetes (using the validated CPCSSN definition), systolic blood pressure (BP), total cholesterol and high density lipoprotein (HDL) cholesterol. Patients with established vascular disease were not included in the FRS calculation, as this score reflects future CVD risk. SES was expressed as a deprivation quintile combining the material and social scores, as described by Pampalon et al., [16] with one (1) being the least deprived and five (5) being the most deprived.

Patients were categorized in the high risk group if any of the following criteria were met:

- FRS ≥ 20%
- Diabetes diagnosis and age ≥ 40 years
- Diabetes diagnosis for ≥ 15 years and age ≥ 30 years
- Low density lipoprotein (LDL) cholesterol ≥ 5.0 mmol/L (at initiation of statin)
- Diagnosis indicating CVD (i.e., secondary prevention), as defined in Appendix A.

Patients deemed to be at moderate risk (that is, FRS between 10–19% and no other criteria from the high-risk group) were not included in the regression analysis because treatment decisions in this group are more nuanced and we did not have information about the clinician-patient conversations around prescribing that may have taken place.

### Secondary outcome measurement

The secondary outcome focused on evaluating the concordance with guideline recommended care for statin prescribing according to CVD risk and SES quintiles. We investigated whether a patient received a statin prescription in accordance with current guidelines by observing the presence of a statin prescription for those considered at high CVD risk or the absence of a statin prescription for those at low CVD risk (FRS < 10% and no high-risk criteria) [2, 9]. Inconsistency with current guidelines was measured by identifying patients who were found to have received a statin prescription and were considered low risk or who were in a high-risk category and did not have evidence of a statin prescription.

Medication records in the EMR reflect products that have been prescribed by primary care providers, but do not necessarily contain all medications prescribed elsewhere (e.g. by a cardiologist). CPCSSN codes prescription data in a standardized way using the Anatomical Therapeutic Chemical (ATC) classification system. The ATC codes used to describe statins and combination medications were C10AA, C10AB, and C10AX. In order to exclude those who did not initiate their medication or who may have been prescribed a statin in error, statin prescription was counted for patients who had at least two refill prescriptions. This method has been used previously [11].

### Analysis

Chi-square was used to determine statistically significant differences between categorical variables, such as age group and deprivation quintiles. A simple logistic regression analysis was used to evaluate the association of patient characteristics on CVD risk. The dependent variable was defined as whether the statin prescription deviated from guideline recommendations (1 = Yes, 0 = No). Independent variables were age group, sex, SES, diabetes and hypertension. A multiple logistic regression analysis was performed using backward elimination on all three independent variables together to adjust for their effects.

SQLServer2012 and Python 2.7.10 were used to retrieve data from CPCSSN and compute the FRS. Statistical analyses were performed using SPSS version 24 and 28 (IBM 2016) [17]. The protocol was reviewed and approved by the Conjoint Heath Research Ethics Board of the University of Calgary (REB17-0992).

## Results

From the initial cohort of patients aged 35–75 years with at least one visit to their primary care provider between 2012–2016 (*N* = 511,610), 324,526 had sufficient information present in their EMR to assign to a CV risk category (Supplementary Fig. 1; Table 1). There was no postal code information for about 45% of these patients, therefore socioeconomic quintiles could only be calculated for 176,787 patients (34.6% of the initial cohort).Table 1 describes the demographic and clinical characteristics of the patients assigned to the three risk categories, by age, sex, FRS and component risk factors, overall CVD risk and SES quintile. A larger proportion of older adults were identified as being at higher CVD risk than younger adults. Males were more often categorized as high risk (48%) compared to females (26%). Individuals who were assigned to the most deprived socioeconomic quintile were more often included in the high-risk category (43%) compared to patients in the least deprived quintile (30%).

**Table 1:** Summary of CVD risk profile for patients (*N* = 324,526)

**Characteristic**	**Overall CVD Risk**			**Total Patients** *N* = 324,526	***p*-value**
	**Low** n (%) *N* = 146,502 (45.1% of total patients)	**Medium** n (%) *N* = 61,077 (18.8% of total patients)	**High** n (%) *N* = 116,947 (36.0% of total patients)		
Age group in years					
35–39	18,038 (12.31)	363 (0.59)	955 (0.82)	19,356 (5.96)	< 0.001
40–44	24,431 (16.68)	1,056 (1.73)	4,565 (3.90)	30,052 (9.26)	
45–49	27,383 (18.69)	3,470 (5.68)	7,363 (6.30)	38,216 (11.78)	
50–54	27,990 (19.11)	8,631 (14.13)	12,407 (10.61)	49,028 (15.11)	
55–59	22,710 (15.50)	12,685 (20.77)	17,838 (15.25)	53,233 (16.40)	
60–64	14,434 (9.85)	13,090 (21.43)	22,366 (19.12)	49,890 (15.37)	
65–69	7,936 (5.42)	11,824 (19.36)	24,743 (21.16)	44,503 (13.71)	
70–75	3,580 (2.44)	9,958 (16.30)	26,710 (22.84)	40,248 (12.40)	
Gender					
Male	42,550 (29.04)	34,631 (56.70)	70,195 (60.00)	147,376 (45.41)	< 0.001
Female	103,952 (70.96)	26,446 (43.30)	46,752 (40.00)	177,150 (54.59)	
Comorbid conditions					
Diabetes Mellitus	1,137 (0.78)	161 (0.26)	61,832 (52.87)	63,130 (19.45)	< 0.001
Hypertension	24,489 (16.72)	23,371 (38.26)	59,903 (51.22)	107,763 (33.21)	< 0.001
Current smoker	9,848 (6.72)	9,884 (16.18)	20,856 (17.83)	40,588 (12.51)	< 0.001
Framingham Risk Score					
Low (< 10%)	146,448 (99.96)	–	15,016 (12.84)	161,464 (49.75)	< 0.001
Med (10–19%)	–	61,119 (100)	26,570 (22.72	87,689 (27.02)	
High (≥ 20%)	–	–	75,373 (64.45)	75,373 (23.23)	
Statin prescription	9,557 (6.52)	12,705 (20.80)	56,130 (48.00)	78,392 (24.16)	< 0.001
Socioeconomic deprivation quintile					
Q1 (least deprived)	20,097 (13.72)	8,105 (13.27)	12,172 (10.41)	40,374 (12.44)	< 0.001
Q2	22,344 (15.25)	8,349 (13.67)	14,038 (12.00)	44,731 (13.78)	
Q3	17,995 (12.28)	6,917 (11.33)	12,224 (10.45)	37,136 (11.44)	
Q4	12,976 (8.86)	5,133 (8.40)	10,686 (9.14)	28,795 (8.87)	
Q5 (most deprived)	10,749 (7.34)	4,005 (6.56)	10,997 (9.40)	25,751 (7.93)	
Missing postal code for SES	62,341 (42.55)	28,568 (46.77)	56,830 (48.59)	147,739 (45.52)	

**Fig. 2:**
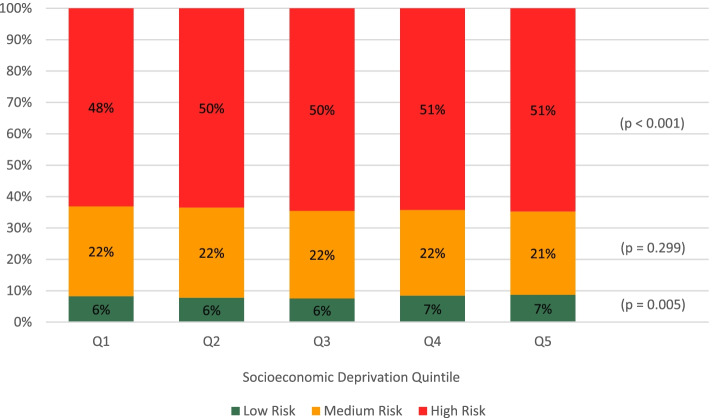
Patients who were prescribed a statin according to CVD risk profile and socioeconomic deprivation quintiles (*N* = 42,360)

When considering statin prescribing guidelines, 7% of low-risk patients had received a statin prescription but only 48% of potentially high-risk patients had received one (Table 1). Figure 2 summarizes the proportion of patients with a statin prescription according to deprivation quintiles and risk groups (*N* = 42,360). Among those in the most deprived quintile, slightly more patients receiving a statin (51%) were categorized as high risk compared to the least deprived quintile (48%) (*p* < 0.001).

The final adjusted logistic model (Table 2) indicated that older patients were more likely to receive statin therapy in a way that was not concordant with the guidelines, relative to a reference age group of 35–44 years. In particular, older patients (aged 65–75 years) were found to have nearly four times the odds of statin prescribing that was not concordant with guidelines (adjusted OR = 3.84, 95% CI 3.66–4.02). Likewise, men have nearly twice the odds as women of receiving statins outside of guideline recommendations for their CVD risk (adjusted OR = 1.74, 95% CI 1.70–1.79). In terms of socioeconomic status, patients in the two most deprived quintiles have 1.10 to 1.21 times the odds of statin prescribing inconsistent with guidelines compared to the least deprived. Patients diagnosed with diabetes and hypertension also had higher odds of being prescribed statins that were not concordant with guidelines, with adjusted OR = 2.25 for those with diabetes and adjusted OR = 1.27 for those with hypertension.

**Table 2:** Assessment of statin prescribing for CVD risk management in accordance with national care guidelines

Characteristic	Statin prescription		Unadjusted^a^		Adjusted^b^	
	Deviates from guidelines	Adheres with guidelines	OR	*p*-value	OR	*p*-value
	N (%)	N (%)	(95% CI)		(95% CI)	
Age group (years)						
35–44	5,157 (10.75)	42,832 (89.25)	reference		reference^c^	
45–54	14,595 (19.42)	60,548 (80.58)	2.00	< 0.001	1.73	< 0.001
			(1.94–2.07)		(1.65–1.82)	
55–64	24,799 (32.06)	52,549 (67.94)	3.92	< 0.001	3.01	< 0.001
			(3.79–4.05)		(2.87–3.15)	
65–75	25,823 (41.01)	37,146 (58.99)	5.77	< 0.001	3.84	< 0.001
			(5.59–5.97)		(3.66–4.02)	
Sex						
Male	38,648 (34.28)	74,097 (65.72)	1.96	< 0.001	1.74	< 0.001
			(1.92–1.99)		(1.70–1.79)	
Female	31,726 (21.05)	118,978 (78.95)	reference	–	reference	–
Socioeconomic deprivation quintiles						
Q1 (least)	7,560 (23.43)	24,709 (76.57)	reference	–	reference	–
Q2	8,436 (23.19)	27,946 (76.81)	0.99	0.456	1.01	0.684
			(0.95–1.02)		(0.97–1.05)	
Q3	7,130 (23.59)	23,089 (76.41)	1.01	0.624	1.01	0.543
			(0.97–1.05)		(0.97–1.05)	
Q4	6,131 (25.91)	17,531 (74.09)	1.14	< 0.001	1.10	< 0.001
			(1.10–1.19)		(1.05–1.14)	
Q5 (most)	6,183 (28.43)	15,563 (71.57)	1.30	< 0.001	1.21	< 0.001
			(1.25–1.35)		(1.16–1.26)	
Diabetes						
Yes	14,383 (43.10)	18,989 (56.90)	3.23	< 0.001	2.25	< 0.001
			(3.15–3.32)		(2.19–2.31)	
No	21,057 (18.99)	89,849 (81.01)	reference	–	reference	–
Hypertension						
Yes	15,335 (35.08)	28,382 (64.92)	2.16	< 0.001	1.27	< 0.001
			(2.11–2.22)		(1.24–1.31)	
No	20,105 (19.99)	80,456 (80.01)	reference	–	reference	–

^a^Based on simple logistic regression models investigating the univariate effects of independent variables on the outcome (deviation from guidelines)

^b^Based on simple logistic regression models investigating the multivariate effects of independent variables on the outcome (deviation from guidelines)

^c^Level of independent variable used as reference against which the odds of the other levels occurring are determined. For example, in this instance, the odds of prescribing outside of guidelines (versus adhering to guidelines) are almost 4 times greater among those aged 65–75 years compared to the 35–44 years group

## Discussion

Among our sample of primary care patients in Canada, 36% were considered to be in a high-risk group, which included a higher proportion of males, older adults, and individuals with lower SES. Our finding that patients who lived in areas associated with high levels of deprivation had an increasingly high-risk CVD profile is not surprising and is consistent with studies in Canada and other international jurisdictions [5, 18–22].

Nearly half of high-risk patients were not receiving statins in accordance with the national guidelines, and few (7%) low-risk individuals were receiving statins when they potentially would not benefit (Table 1). Of concern, there was a clear gradient in the prescribing of statins in accordance with guidelines by SES, with patients in the most deprived quintile at higher odds of experiencing CVD management that deviated from clinical guidelines. Similarly inequitable statin prescribing patterns by socioeconomic status were also observed in Denmark, although only among men [23]. The reasons for this are multifaceted and often interlinked; for example, education, occupation and income are consistently associated with many cardiovascular risks, including smoking, hypertension, obesity, physical inactivity, and poor diet, among others [5]. In addition, lower SES is also correlated with poor health literacy and medication non-compliance, resulting from lower levels of education and an inability to afford prescription medications [5, 24, 25]. The effect of social determinants on health and medical care is apparent here and in many other examples worldwide [26, 27].

We anticipate this study will provide an opportunity for clinical improvement in primary care settings. As practices in Canada move toward implementing models of care focused on the patient’s "medical home", [28] strategies to ensure the consistent collection of relevant risk data, including social determinants of health, is an important first step. The subsequent use of these clinical and social risk data to improve patient outcomes is challenging, especially given limited clinician time and guidance, as well as a potential lack of available and appropriate social resources [29]. However, there are examples of ongoing efforts to promote equity-focused primary health care that exist in Canada [30–32], with the College of Family Physicians of Canada strongly advocating for health equity as part of their recent strategic plan [33].

### Limitations

While this analysis used a large, pan-Canadian sample of primary care patients, the CPCSSN dataset only includes individuals who sought care from their primary care provider. Thus, it may be more likely to contain information for patients who are more ill or visit their provider more often. Our sample was also hindered by missing data; for instance, 45% of patients were missing full postcode required to calculate the Pampalon deprivation quintiles. Some contributing CPCSSN networks do not provide full postal codes for various technical or procedural reasons. It is important to note that the Pampalon index of material and social deprivation is an ecological measure based on the postcode of residence and does not directly reflect the SES of individuals. Further, many individuals were missing data related to their FRS and therefore, the absolute number of patients at risk is likely underestimated. Missing data is a common challenge for analyses using EMR data and of the variables used in the FRS, patient smoking status is most often underreported in primary care EMR data, despite its importance as a risk factor for CVD [34–37].

The 2016 CCS guidelines used in this study were published at the end of the study time period (2012–2016) and may not have reflected previously recommended prescribing practices. However, there was little difference between the updated 2016 guidelines and its predecessor published in 2012, particular for the high-risk groups.

Lastly, there is insufficient information in the EMR to discern why these prescribing decisions were made or whether treatment was offered but declined. We did not report on the presence of chronic kidney disease (CKD) due to the absence of a validated definition for CPCSSN data at the time of this analysis, which may have been the rationale for statin prescribing when it was seemingly unwarranted. Even so, most patients with CKD experience other vascular risk factors, such as diabetes and hypertension, which would have been captured.

## Conclusions

In Canada, primary care patients who are considered at high CVD risk are more often male, older, current smokers, in a lower socioeconomic quintile and with a higher prevalence of comorbid hypertension or diabetes, compared to those at low CVD risk. The odds of receiving care that was not concordant with current care guidelines were found to be greater in the most deprived group. A focus on comprehensive, systematic documentation of CVD risk factors in clinical practice, with attention to socioeconomic influences, may provide action-oriented risk assessment for patients at higher risk of CVD.

## Supplementary information

**Additional file 1:** Appendix A: ICD**-**9 codes used to identify patients in the secondary prevention group

**Additional file 2:** The RECORD statement – checklist of items, extended from the STROBE statement, that should be reported in observational studies using routinely collected health data

**Additional file 3:** Patient flow diagram

## Data Availability

The de-identified data that support the findings of this study are available from the national CPCSSN repository but restrictions may apply to the availability of these data, which were used under strict data security and privacy protocols, and so are not publicly available. Access to the national CPCSSN database is however available to approved researchers according to the CPCSSN data access guidelines found here: http://cpcssn.ca/dar/

## Abbreviations

ATC: Anatomical Therapeutic Chemical [classification system]
BP: Blood pressure
CCS: Canadian Cardiovascular Society
CKD: Chronic kidney disease
CPCSSN: Canadian Primary Care Sentinel Surveillance Network
CVD: Cardiovascular disease
EMR: Electronic medical record
FRS: Framingham risk score
HDL: High density lipoprotein
LDL: Low density lipoprotein
mmol/L: Millimole per litre
OR: Odds ratio
SES: Socioeconomic status
