# Supplementary material for: A cross-sectional study evaluating cardiovascular risk and statin prescribing in the Canadian Primary Care Sentinel Surveillance Network database
Source: BMC Prim Care. 2022 May 25;23:128. doi: 10.1186/s12875-022-01735-6 (PMC9131688; doi:10.1186/s12875-022-01735-6)
Supplement: Supplementary file 1 — Additional file 1: Appendix A: ICD-9 codes used to identify patients in the secondary prevention group [file 12875_2022_1735_MOESM1_ESM.pdf]

Appendix A: ICD-9 codes used to identify patients in the secondary prevention group

Code	Diagnosis
410	Ischaemic Heart Disease
411	
412	
413	
414	
424	Other diseases of endocardium
425	Cardiomyopathy
428	Heart Failure
429.2	Cardiovascular disease unspecified
429.9	Heart Disease unspecified
433	Occlusion and stenosis of precerebral arteries
434	Occlusion of cerebral arteries
435	Transient Cerebral ischaemia
436	Acute but ill defines cerebrovascular disease
437	Other and ill defined cerebrovascular disease
438	Late effects of cerebrovascular disease
440	Atherosclerosis
441	Aortic aneurysm and dissesction
442	Other aneurysm
443	Other peripheral vascular disease
444	Arterial embolism and thrombosis
445	Atheroembolism
