# Supplementary material for: A cross-sectional study evaluating cardiovascular risk and statin prescribing in the Canadian Primary Care Sentinel Surveillance Network database
Source: BMC Prim Care. 2022 May 25;23:128. doi: 10.1186/s12875-022-01735-6 (PMC9131688; doi:10.1186/s12875-022-01735-6)
Supplement: Supplementary file 3 — Additional file 3: Patient flow diagram [file 12875_2022_1735_MOESM3_ESM.pdf]

Supplementary File: Patient flow diagram.

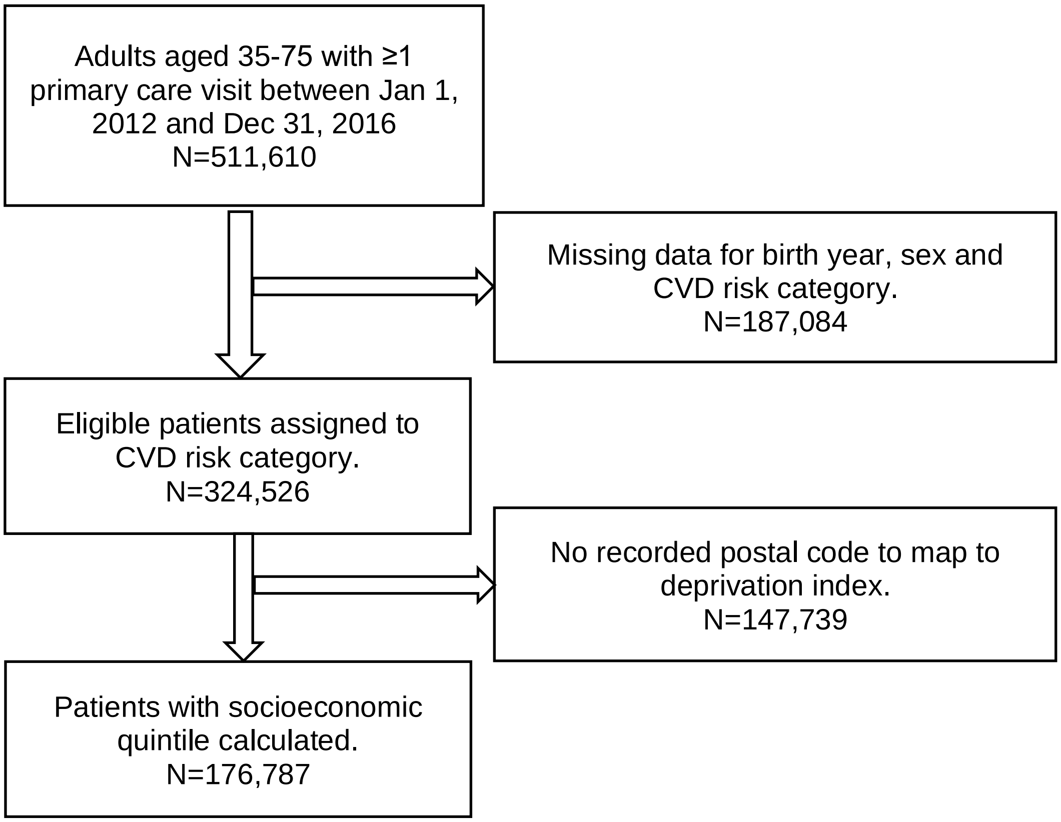

NB: high, moderate and low CVD risk categories determined according to the 2016 Canadian Cardiovascular Society guidelines.
